# Nicotinamide Riboside Attenuates Memory Impairment and Depressive-like Behavior in an Alzheimer’s Disease Animal Model

**DOI:** 10.1192/j.eurpsy.2024.196

**Published:** 2024-08-27

**Authors:** A. Aytulun, B. Cimen, Y. Sara, S. Ö. Erden Aki

**Affiliations:** ^1^Psychiatry, Kilis Prof Dr Alaeddin Yavaşça State Hospital, Kilis; ^2^Medical Pharmacology; ^3^Psychiatry, Hacettepe University Faculty of Medicine, Ankara, Türkiye

## Abstract

**Introduction:**

Depression in Alzheimer’s disease (AD) differs from major depression in terms of clinical features and treatment. Antidepressants do not provide the expected benefits in depressive symptoms accompanying cognitive decline in AD, suggesting distinct mechanisms. Emerging research suggest that compromised mitophagy, the selective removal of damaged mitochondria, may contribute to the pathogenesis of AD. However boosting nicotinamide adenine dinucleotide (NAD+) to induce mitophagy reduces amyloid β (Aβ) aggregation and enhances cognitive function in AD models (Kerr *et al*.,Trends Neurosci 2017;40:151-66). Nevertheless, data on NAD’s impact on depression in AD remains limited.

**Objectives:**

This study aimed to examine the impact of the NAD+ precursor nicotinamide riboside (NR) on cognitive and neuropsychiatric symptoms in a AD rat model.

**Methods:**

To induce the AD, a single dose of 5 μl Aβ1-42 was injected into each lateral ventricle of rats (day 0), while the control group received an intracerebroventricular (icv) saline (0.9%NaCl).Four experimental groups were designed: control (icv saline+po saline), NR (icv saline+po NR), Aβ (icv Aβ+po saline), and Aβ+NR (icv Aβ+po NR).After the injection, to reduce Aβ clearance (Kang *et al.* Science. 2009;32 1005-7.) rats were subjected to 96 hours of sleep deprivation.Starting from day 6, rats were given either 700 mg/kg oral NR or saline, and handling test scores were recorded daily.The procedures were repeated daily until the rats were sacrificed on day 28.Behavioral experiments were randomly conducted at the end, and statistical analysis was performed using repeated measures ANOVA, followed by the Tukey post hoc test.

**Results:**

Passive avoidance test results showed that the Aβ group had the shortest latency to enter the dark area. However, the Aβ+NR group exhibited a prolonged latency compared to the Aβ group (F(3,2)=5.5;p<0.05). Aβ injection induced depressive-like behavior in rats, as indicated by the forced swim test (FST) for behavioral despair and the sucrose preference test (SPT) for anhedonia. In AD rats treated with NR (Aβ+NR), Aβ-induced depressive-like behavior was reduced, with lower FST immobility scores (F(3,2)=6.2;p<0.05) and increased sucrose preference in the SPT (F(3,2)=7.5;p<0.05). There were no significant differences in anxiety-like behaviors among the groups, assessed by the time spent in the open arm in the elevated plus maze test (F(3,2)=1.9;p>0.05). During the 28-day monitoring period, the Aβ+NR group of rats exhibited a more rapid decrease in aggression levels compared to the other groups in the handling test. This decrease was significant between days 7 and 10 compared to the Aβ group (F(48,5)=1.5;p<0.05).

**Conclusions:**

NR improved memory, reduced depressive behavior, and lowered aggression in AD rats. This suggests that NAD+ precursor NR effectively treats cognitive decline and neuropsychiatric symptoms in an AD model.

**Disclosure of Interest:**

None Declared

